# Thematic Selection: Stress and Stress-related Disorders Posttraumatic Stress Disorder (Part 2)

**DOI:** 10.2174/1570159X2204231106143917

**Published:** 2023-11-06

**Authors:** Agorastos Agorastos

**Affiliations:** 1 Assistant Professor of Psychiatry, Aristotle University of Thessaloniki, Greece

The history of humanity is marked by violence, abuse, war and disasters, all of which have had a devastating impact of trauma on individuals, families, communities and societies. From a philosophical standpoint, trauma illuminates a deep existential dilemma between human fragility and resilience, health and disease, and the quality of life and death. Psychological and physical consequences of trauma have been reported from mankind’s first Epic of Gilgamesh to Homer’s Iliad, while several terms have been coined to describe them (*e.g*., cardiorespiratory neurosis, railway spine, shell shock, war neurosis, soldier’s heart, post-Vietnam syndrome, *etc*.) [1]. In 1952, DSM-I proceeded to the formulation of the diagnostic category “gross stress reaction” for the symptom cluster seen after a traumatic event and in 1980, DSM-III adopted, for the first time, the term posttraumatic stress disorder (PTSD) after the increasingly diagnosed posttraumatic symptoms in the Vietnam veterans [1].

PTSD is a chronic and debilitating condition of relatively high lifetime prevalence and disease burden that manifests in the aftermath of one or more profoundly traumatic or life-threatening experiences [2-4]. The diagnosis of PTSD requires at least one month of symptoms (Criterion F) following exposure to an actual, threatened or witnessed major traumatic event outside the range of usual human experience (Criterion A) [5, 6]. Typical signs include reexperiencing/intrusion symptoms (Criterion B), avoidance behaviors (Criterion C), cognitive and emotional symptoms (Criterion D), increased arousal and physiologic symptoms (Criterion E) and functional impairment (Criterion G). PTSD, however, exhibits distinct clinical phenotypes such as classic, dissociative, and complex presentations. It also displays variations in the timing of onset, along with diverse quantitative and qualitative course developments. Frequently, it includes nonspecific subsyndromal symptoms or overlapping symptoms with other diagnostic categories like depression, anxiety disorders, and insomnia. Thereby, individual susceptibility or resilience factors (*e.g*., personality traits, attachment style, support system, self-efficacy, marital and occupational status), as well as other trauma-related characteristics (*e.g*., severity, type of trauma, time of day), peritraumatic reactions, but also the history of repeated or continuous exposure can influence the individual development of traumatic sequalae at clinical level [3, 7-9].

Traumatic stress exposure has been proven to lead to sustained alterations in stress regulation and psychophysiological reactivity, impaired glucocorticoid signaling, changes in hypothalamic–pituitary–adrenal axis and sympathetic–adrenal–medullary system, as well as to epigenetic modifications [9, 10]. This can gradually affect master homeostatic systems at the crossroads of peripheral and central susceptibility pathways, leading to the biological embedment of the traumatic stress through neurobiological alterations with profound, cumulative and debilitating effects for health, overall disease vulnerability, physical and mental co-morbidity, as well as all-cause mortality even decades later [9, 11-17].

This current issue (Part 2) of the special thematic selection entitled “Stress and stress-related disorders” offers a comprehensive collection of articles on PTSD from molecular pathophysiology to cutting edge treatments.

With their article, Antolasic *et al.* review theoretical underpinnings of the involvement of BDNF and the Val66Met polymorphism in the development and persistence of intrusive and hypervigilance symptoms in PTSD [18]. Patas *et al.* provide a novel take on immune and inflammatory findings in PTSD and offer some stimulating ideas on immune-based PTSD treatment alternatives [19]. The article by Sabé *et al.* embodies a large scientometric analysis of 42,170 publications published between 1945 and 2022 to outline different aspects of clinical research in PTSD [20], while Lappas *et al.* offer a detailed review of available literature on the effect of antidepressants on sleep in patients with PTSD [21]. Finally, this special issue includes two seminal articles by two world-expert research groups in PTSD. The first paper by Burback *et al.* comes from Professor Vermetten’s research group in Leiden NL, and offers a very large, state-of-the-art comprehensive review on pharmacological, psychotherapeutic, behavioral and technology-related treatment of PTSD, which also initially provides an expert overview on symptomology, diagnostic issues, neurobiology and risk factors of PTSD and includes almost 1,000 references [22]. The second seminal paper by Zaretsky *et al.* comes from Professor Yehuda’s research group at Mount Sinai in New York, USA, and offers a state-of-the-art comprehensive review on the data supporting the current and future use of psychedelics in the treatment of PTSD while also presenting history of use, pharmacology and safety profile of all reviewed substances and includes almost 900 references [23].

In reflecting upon the profound research, clinical, individual and societal challenges of PTSD, one can only note an urgent current need to deepen our understandingof the neurobiological trajectories of PTSD development and course towards a chronic systematic disease continuum model and, herewith, better provide established treatments and also recognize new potentials for patients in need according to individual symptoms and risk/resilience factors.
